# Virtual Reality Intervention for Patients With Neck Pain: Systematic Review and Meta-analysis of Randomized Controlled Trials

**DOI:** 10.2196/38256

**Published:** 2023-04-03

**Authors:** Qifan Guo, LIMing Zhang, Chenfan Gui, Guanghui Chen, Yi Chen, Huixin Tan, Wei Su, Ruishi Zhang, Qiang Gao

**Affiliations:** 1 Department of Rehabilitation Medicine West China Hospital Sichuan University Chengdu China; 2 Department of Traumatology and Orthopedics of Traditional Chinese Medicine The First Affiliated Hospital of Guangxi University of Chinese Medicine Nanning China

**Keywords:** meta-analysis, virtual reality, neck pain, disability, systematic review

## Abstract

**Background:**

Neck pain is a prevalent condition that causes an enormous health care burden due to the lack of efficient therapies. As a promising technology, virtual reality (VR) has shown advantages in orthopedic rehabilitation. However, there is no meta-analysis evaluating the effectiveness of VR in neck pain management.

**Objective:**

This study aims to review original randomized controlled trials (RCTs) evaluating the effectiveness of VR for neck pain and to provide evidence for the clinical application of a new alternative approach for pain management.

**Methods:**

A total of 9 electronic databases were systematically searched for relevant articles published from inception to October 2022. RCTs in English or Chinese that investigated VR therapy for participants with neck pain were included. The methodological quality and the evidence level were assessed using the Cochrane Back and Neck Risk of Bias tool and the Grading of Recommendations Assessment, Development, and Evaluation (GRADE) guideline, respectively.

**Results:**

A total of 8 studies with 382 participants were included for the final analysis. For the pain intensity, the overall pooled effect size was 0.51, with a standardized mean difference (SMD) of −0.51 (95% CI −0.91 to −0.11; GRADE: moderate), favoring VR therapy compared with controls. Subgroups analyses revealed that significant differences in pain intensity were found in the multimodal intervention (VR in combination with other therapies) than in other interventions (SMD −0.45, 95% CI −0.78 to −0.13; GRADE: moderate), and better analgesic effects were also observed in patients with chronic neck pain receiving VR intervention (SMD −0.70, 95% CI −1.08 to −0.32; GRADE: moderate) and patients treated in the clinic or research unit (SMD −0.52, 95% CI −0.99 to −0.05; GRADE: moderate) than controls. Regarding other health outcomes, the VR experienced less disability, lower kinesiophobia, and greater kinematic function (cervical range of motion, mean and peak velocity). Nevertheless, the follow-up effects of VR therapy on pain intensity and disability were not found.

**Conclusions:**

Existing moderate evidence support VR as a beneficial nonpharmacological approach to improve pain intensity in patients with neck pain, with advantages to multimodal intervention, people with chronic neck pain, and clinic or research unit–based VR therapy. However, the limited quantity and high heterogeneity of the articles limit our findings.

**Trial Registration:**

PROSPERO CRD42020188635; https://tinyurl.com/2839jh8w

## Introduction

Neck pain is a worldwide condition, with nearly 60% to 80% of individuals developing neck pain during their lifetime [1,2]. Most patients with neck pain experience various physical impairments, such as reduced cervical range of motion (CROM) and moving speed [3,4]. In addition, neck pain can lead to various psychological issues (eg, fear of movement and depression) [5,6]. These issues may impair patients’ work performance and quality of life, leading to large economic losses [7]. Current treatments for this health condition are mainly medications, surgeries, and conservative therapies (eg, physiotherapy or acupuncture), which can be time-consuming, expensive, and unsustainable [8]. Therefore, there is an urgent need to explore effective treatments for patients with neck pain.

Exercise is recommended by current clinical guidelines as an effective treatment for patients with neck pain [9,10]. Virtual reality (VR) is a unique form of exercise established by Morton Heiling in 1962 and has been evolving over the past 60 years [11,12]. VR technology is defined as a system that allows users to interact with images and sounds in a virtual environment, which can stimulate response and provide real-time feedback concerning their performance. This technology can be combined with computer or mobile device screens and head-mounted displays to better interact with users [13,14].

Over the past decade, VR has gradually become a valuable tool for assessment and intervention in clinical rehabilitation due to the continuous research and cost reduction in the field of virtual technology [15]. A typical example of the application of VR in the medical field is neurological rehabilitation, especially after a stroke [16,17]. Numerous studies [18-20] have shown that VR therapy can greatly improve upper limb motor function and cognitive abilities in people who have had a stroke with an acceptable safety profile. Other benefits of VR therapy could be realized, on the other hand, in the management of patients with mental health disorders, such as anxiety, depression, drug addiction, and eating disorders [21,22]. The potential therapeutic mechanisms of VR include task-oriented repetition, positive feedback, and embodied simulation [23]. In addition, VR can also assist researchers and clinicians in data collection and monitoring of therapeutic processes via related evaluation tools, which can facilitate medical decision-making and enhance safety in clinical practice [24,25].

As a noninvasive analgesic approach, VR therapy has attracted plenty of studies on pain management. Previous studies [26-28] demonstrated the potential efficacy of VR-based rehabilitation on pain and disability in individuals with orthopedic diseases, including rheumatoid arthritis, shoulder impingement syndrome, low back pain, and chronic neck pain. In addition, a systematic review [29] concluded that VR could improve pain intensity and disability compared to other interventions in patients with neck or lower back pain. However, to our knowledge, no meta-analysis has been carried out to critically evaluate the intervention effects of VR on neck pain. Therefore, we aimed to conduct a meta-analysis of randomized controlled trials (RCTs) through multiple literature searches to investigate the potential efficacy of VR in reducing pain intensity in patients with neck pain.

## Methods

**Study Protocol and Registration**

This study protocol was registered on PROSPERO (CRD42020188635). This study was reported according to the PRISMA (Preferred Reporting Items for Systematic Reviews and Meta-analyses) guidelines to ensure the transparency of the research [30]. The Cochrane Handbook for Systematic Reviews of Interventions (version 5.1.0) was followed [31].

**Data Sources and Searches**

Databases utilized to search the eligible trials include 7 English literature databases, namely, Medline (via PubMed), Embase, Web of Science Core Collection, CENTRAL, Scopus, Physiotherapy Evidence Database (PEDro), and ClinicalTrial, as well as 2 Chinese literature databases, namely, China National Knowledge Infrastructure Library and Wan Fang database. The databases were searched from their inception until October 2022. Relevant journals were manually searched to identify eligible studies. The last search was conducted on October 30, 2022.

The search was performed using a combination of relevant Medical Subject Headings (MeSH) terms and free text words: (neck pain **or** neck ache **or** cervical spondylosis) AND (virtual reality **or** virtual reality exposure therapy **or** VR **or** virtual reality simulator **or** virtual reality system **or** virtual reality head-mounted display **or** telerehabilitation **or** remote rehabilitation). Search strategies for each database are presented in Multimedia Appendix 1. After the selection stage, a further search was carried out by tracking the citations of the included trial (snowballing). The inclusion and exclusion criteria of studies were designed based on the PICO (Participants, Interventions, Control, and Outcomes) principle [32].

### Study Selection

Studies were included for RCTs reported in English or Chinese and published in a peer-reviewed journal. The selection criteria were established according to the prespecified PICO strategy: (1) Participants: patients with neck pain, irrespective of age and the stage of pain; (2) Interventions: unimodal intervention (VR therapy alone) or multimodal intervention (VR therapy in combination with other interventions), including various VR delivery device and levels of immersion. We define VR therapy as a technology that enables patients to interact with a virtual environment by motion sensors or other devices and receive real-time feedback to improve their performance; (3) Control: comparison with other interventions (eg, interventions without VR, standard treatment, no intervention); (4) Outcomes: pain intensity and other health outcomes related to neck pain.

Studies were excluded if they were nonrandomized controlled trials or quasi-RCTs, where quasi-randomized was considered as allocating patients based on a pseudorandom sequence (eg, admission number, date of birth, or alternate assignment). In addition, clinical observations, case reports, letters, abstracts, review articles, studies published in languages other than English and Chinese, and those with insufficient data after contacting the author were excluded from the final synthesis.

### Outcome Measures

Pain intensity measured by a numeric rating scale (NRS) or a visual analog scale (VAS) was the primary outcome, and disability, kinesiophobia, CROM, and motion velocity (mean and peak) were descriptively presented as the secondary outcomes.

### Identification of Studies

Duplicates were removed by EndNote X9 (Clarivate Analytics), and then 2 reviewers (authors QFG and LZ) independently screened the titles, abstracts, and full texts within the included databases to identify the relevant studies. Any discrepancies were resolved by discussion or by consulting a third reviewer (author QG).

### Data Extraction and Management

The extracted data included basic information about the study (ie, author name, year published, and country); risk of bias based on the Cochrane Back and Neck (CBN) Risk of Bias tool [33]; patients’ demographic information (ie, sample size, age, sex ratio, and symptoms duration); type of intervention (ie, brief details of VR therapy, duration, and the number of sessions); type of control; outcomes (ie, time of outcome assessment, and outcome measures); and adverse events. In addition, follow-up data were also collected to present the lasting effect of VR therapy. Where available, mean and SD values were extracted from the text and tables.

Two reviewers (authors QFG and LZ) conducted data extraction independently based on the predecided data extraction form. Any dispute was solved by consulting the third reviewer (author QG). If the relevant data were not enough, we contacted the original author for more information via email.

### Quality Assessment

The 2 reviewers (authors QFG and LZ) independently evaluated the methodological quality and the evidence levels of the included trials using the updated 2015 CBN Risk of Bias tool [33] and the Grading of Recommendations Assessment, Development, and Evaluation guideline (GRADE) [34], respectively. Unresolved disagreements were reviewed by the third reviewer (author QG).

The Risk of Bias tool is recommended by the CBN group for quality assessments of studies on neck or back pain and has demonstrated great interrater reliability [35]. It consists of 13 items in the following domains: randomization, concealed allocation, blinding (participants, personnel, and assessor), intention-to-treat, dropouts, reporting bias, baseline differences, cointerventions, compliance, timing, and other bias. Data were imported into RevMan (version 5.3; Cochrane Collaboration) software to create the risk-of-bias plots.

The GRADE guidelines were used to assess the certainty of the evidence for each primary and secondary outcome measure in the meta-analysis [36]. This grading criterion classified the evidence into 4 levels (ie, high, moderate, low, and very low) depending on the bias factors, including the risk of bias, inconsistency, indirectness, imprecision, and other considerations.

### Statistical Analysis

The aforementioned RevMan software was used to perform the statistical analysis and create forest plots to display the results. Related statistical indicators (mean, SD, and sample size) were extracted and imported into RevMan. Continuous outcomes were presented using mean difference for outcomes measured using the same instrument, standardized mean difference (SMD) for outcomes measured by different methods, and 95% CIs. A fixed effects model was used to calculate the size of the pooled effect. When significant heterogeneity (*I*²>50%) was observed, the random effects model was used, and subgroup analysis was conducted to explore the possible causes of heterogeneity among the studies. Subgroups analyses were performed according to the comparisons of intervention (unimodal vs multimodal intervention), the stage of neck pain (chronic neck pain vs various stages including acute, subacute, and chronic neck pain), the clinical operational model of VR therapy (clinic or research unit–based therapy vs home-based therapy), and the type of scale used (VAS vs NRS).

Regarding the follow-up results, only follow-up effects on pain intensity and disability were explored due to the lack of current studies.

## Results

### Search and Selection

**Figure 1:**
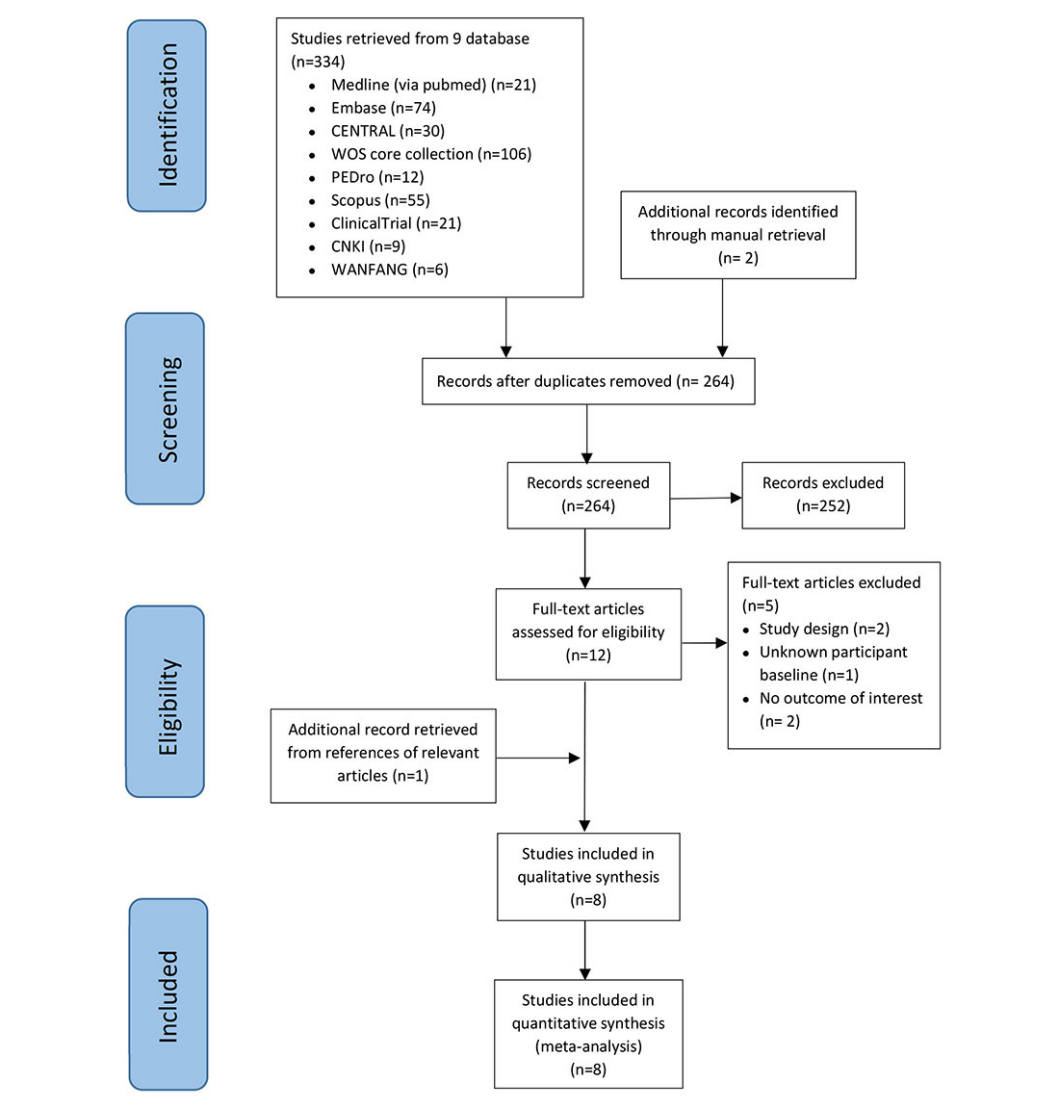
Flow chart of the review process.

A total of 334 records were selected from 7 English and 2 Chinese electronic databases. Two studies were obtained through manual retrieval. After removing duplicates, 264 studies remained, among which 12 studies were identified for full-text retrieval based on the aforementioned criteria. An additional article [37] was retrieved through the references of relevant articles, yielding a sum of 8 studies [37-44]. All 8 studies were included in the final quantitative synthesis. Figure 1 presents the selection process and reasons for study exclusion.

### Study and Patient Characteristics

#### Study Characteristics

All 8 (100%) RCTs [37-44] included in the meta-analysis were written in English. They were conducted in Oceania (Australia [38,42]), Europe (Spain [39], Germany [40], and Turkey [44]), and Asia (Iran [41], India [37], and Israel [43]). The studies were published between 2015 and 2022, and a total of 382 participants (intervention: 167; control: 215) were enrolled. The sample sizes of these studies ranged from 32 to 90. Detailed characteristics of the eligible studies are shown in Table 1.

**Table 1:** Summary of the included studies.

Author, year	Patient characteristics				Intervention			Dosage		Outcomes		Time points
	Participants, n (F^a^/M^b^)	Age (years), mean (SD)	Stage of pain	Experiment		Control						
Sarig Bahat et al [38], 2015	32 (21/11)	IG^c^ (n=16): 40.63 (14.18); CG^d^ (n=16): 41.13 (12.59)	Chronic neck pain	IG: kinematic training + VR^e^ therapy		CG: kinematic training	4-6 sessions for 30 min each week over 5 weeks		VAS^f^, NDI^g^, TSK^h^, CROM^i^, and velocity (mean and peak)		Preintervention, postintervention (5 weeks), and follow-up (3 months)	
Tejera et al [39], 2020	44 (23/21)	IG (n=22): 32.72 (11.63); CG (n=22): 26.68 (9.21)	Chronic neck pain	IG: VR therapy		CG: exercise	8 treatment sessions for 4 weeks		VAS, NDI, TSK		Preintervention, postintervention (4 weeks), follow-up (1 month), and follow-up (3 months)	
Nusser et al [40], 2021	51 (32/19)	IG (n=17): 51.2 (8.8); CG1 (n=16): 53.1 (5.7); CG2 (n=18): 49.8 (8.1)	Chronic neck pain	IG: VR therapy + standard rehabilitation		CG1: Sensorimotor training + standard rehabilitation CG2: standard rehabilitation	6 20-min sessions over 3 weeks		NRS^j^, NDI, CROM		Preintervention and postintervention (3 weeks)	
Rezaei et al [41], 2015	42 (20/22)	IG (n=21): 36.19 (9.80); CG (n=21): 31.23 (9.49)	Chronic neck pain	IG: VR therapy		CG: conventional proprioceptive training	8 training sessions over 4 weeks		VAS, NDI		Preintervention, postintervention (4 weeks), and follow-up (5 weeks)	
Sarig Bahat et al [42], 2017	90 (63/27)	IG (n=30): 48 (NR^k^); CG1 (n=30): 48 (NR); CG2 (n=30): 48 (NR)	Chronic neck pain	IG: VR therapy		CG1: laser exercise CG2: no intervention	20 min a day, 4 times a week, for 4 weeks		VAS, NDI, TSK, CROM, velocity (mean and peak)		Preintervention, postintervention (4 weeks), and follow-up (3 months)	
Mukherjee et al [37], 2021	44 (21/23)	IG (n=22): 55.81 (15); CG (n=22): 54.81 (13)	Subacute or chronic neck pain	IG: VR therapy + conventional physiotherapy		CG: conventional physiotherapy	10 min each day for 3 consecutive days for 1 week		NRS, TSK, CROM		Preintervention and postintervention (3 days)	
Sarig Bahat et al [43], 2020	45 (4/41)	IG (n=22): 30 (5.8); CG (n=23): 28 (5.1)	Acute, subacute, or chronic neck pain	IG: VR therapy		CG: conventional physical therapy	20 min for each week over 4 weeks		VAS, NDI, ROM, velocity (mean and peak)		Preintervention, postintervention (4 weeks), and follow-up (6 months)	
Cetin et al [44], 2022	34 (23/11)	IG (n=17): 40 (11.88); CG (n=17): 41.94 (10.76)	Chronic neck pain	IG: VR therapy + Motor control		CG: Motor control	40 min each session for 18 sessions over 6 weeks		VAS, CROM		Preintervention and postintervention (6 weeks)	

^a^F: female.

^b^M: male.

^c^IG: intervention group.

^d^CG: control group.

^e^VR: virtual reality.

^f^VAS: visual analog scale.

^g^NDI: Neck Disability Index.

^h^TSK: Tampa Scale of Kinesiophobia.

^i^CROM: cervical range of motion.

^j^NRS: numeric rating scale.

^k^NR: not reported.

#### Participant Characteristics

The 8 studies [37-44] included participants with chronic neck pain, among which 1 (13%) study [37] also included patients in the subacute phase, and another study (n=1, 13%) [43] recruited patients in the acute or subacute stages. All studies included both male and female participants, and 5 (63%) [38,39,42-44] included more females than males. The mean age of patients ranged between 26.68 (SD 9.21) years and 55.81 (SD 15) years. Only 2 (25%) studies [38,41] reported the duration of symptoms, which ranged from 22.04 (SD 16.79) months to 98.06 (SD 96.81) months. The characteristics of the participants are presented in Table 1.

#### Intervention

The 8 studies [37-44] compared VR with other interventions (eg, kinematic exercise [38,39], general sensorimotor training [40], conventional rehabilitation [37,40,43], proprioceptive training [41], laser training [42], motor control [44], and no intervention [42]). All interventions used the immersive VR device, with the intervention duration varying from 1 to 6 weeks and intervention frequency ranging from once per week to once per day. As a traditional face-to-face care model, participants in 7 (88%) studies [37-41,43,44] received VR therapy in a clinic [37,40,44] or research unit [38,39,41,43]. However, 1 (13%) study [42] adopted a new approach to telemedicine and home-based rehabilitation.

Various VR programs were developed in the included studies. To reduce disability, Sarig Bahat et al [38] from Australia developed a VR system with 3 modules containing CROM, velocity, and accuracy therapy, which were tailored to each participant and progressed according to their performance. Participants were guided to complete between 4 and 6 supervised intervention sessions over 5 weeks. Similarly, Tejera et al [39] from Spain used a VR program that allowed participants to perform cervical flexion, extension, rotation, and lateral flexion movements when immersed in a simulated living room or ocean. The photos of animals in the simulated environment offered enough feedback to motivate the participants’ neck motions. The participants were recommended to perform 3 series comprising 10 repetitions of VR exercise with 30 seconds of rest between exercises. Nusser and colleagues [40] from Germany provided VR-based “neck-specific sensorimotor training” for participants with nontraumatic chronic neck pain. During treatment, participants were asked to gradually follow a virtual globe by increasing the CROM to train their cervical kinematic function. The training was divided into six 20-minute sessions, during which the study staff provided assistance. In Iran, Rezaei and colleagues [41] studied the effectiveness of VR (Cervigame) for adults with neck pain. The novel video game comprised 50 stages that were further divided into unidirectional and 2-directional stages ranging from easy to hard. Participants were required to complete 8 training sessions over 1 month. In Australia, Sarig Bahat and colleagues [42] had participants with chronic neck pain receive VR training at home 4 times each week for 1 month. Each participant was provided with a training plan directed toward (1) increasing CROM, (2) increasing motion velocity, and (3) increasing motion accuracy in smooth head pursuit, which was also applied in another study conducted in Israel [43]. Mukherjee et al [37] from India conducted a VR therapy using an immersive VR headset. Participants were requested to sit on a chair with back support and move their necks to hit each virtual goal by increasing their CROM for 3 consecutive days a week. In addition, the VR equipment applied by Cetin et al [44] from Turkey enabled the participants to sit in a chair that allowed 360° movement and required them to rotate their necks in all directions during VR sessions to achieve therapeutic effects. Patients in the intervention group were expected to receive 20 minutes of VR treatment each session and attend a total of 18 treatments over 6 weeks. The characteristics and details of each intervention are listed in Table 1.

#### Outcome Measures

Various instruments were used to measure the intervention effects. For the primary outcomes, pain intensity was measured using a VAS [38,39,41-44] and an NRS [37,40]. These 2 tools graded the pain intensity from 0 (no pain) to 10 cm or 100 mm (worst pain imaginable) [45-47]. For the secondary outcomes, disability was evaluated by the Neck Disability Index (NDI) in 5 (63%) studies [38-42]. Kinesiophobia was assessed using the Tampa Scale of Kinesiophobia (TSK) in 4 (50%) studies [37-39,42]. Additionally, 5 (63%) studies evaluated CROM using VR devices [38,40,42], a cervical measuring gauge [44], and a standard goniometer [37], respectively. Two (25%) studies [38,42] measured the mean and peak velocity of cervical motion by VR devices. These tools (NDI, TSK, VR devices, and standard goniometer) have proven to have high validity and reliability in measuring these health indicators [48-51]. The outcome measures are shown in Table 1.

### Quality and Certainty of Evidence Assessment

**Figure 2:**
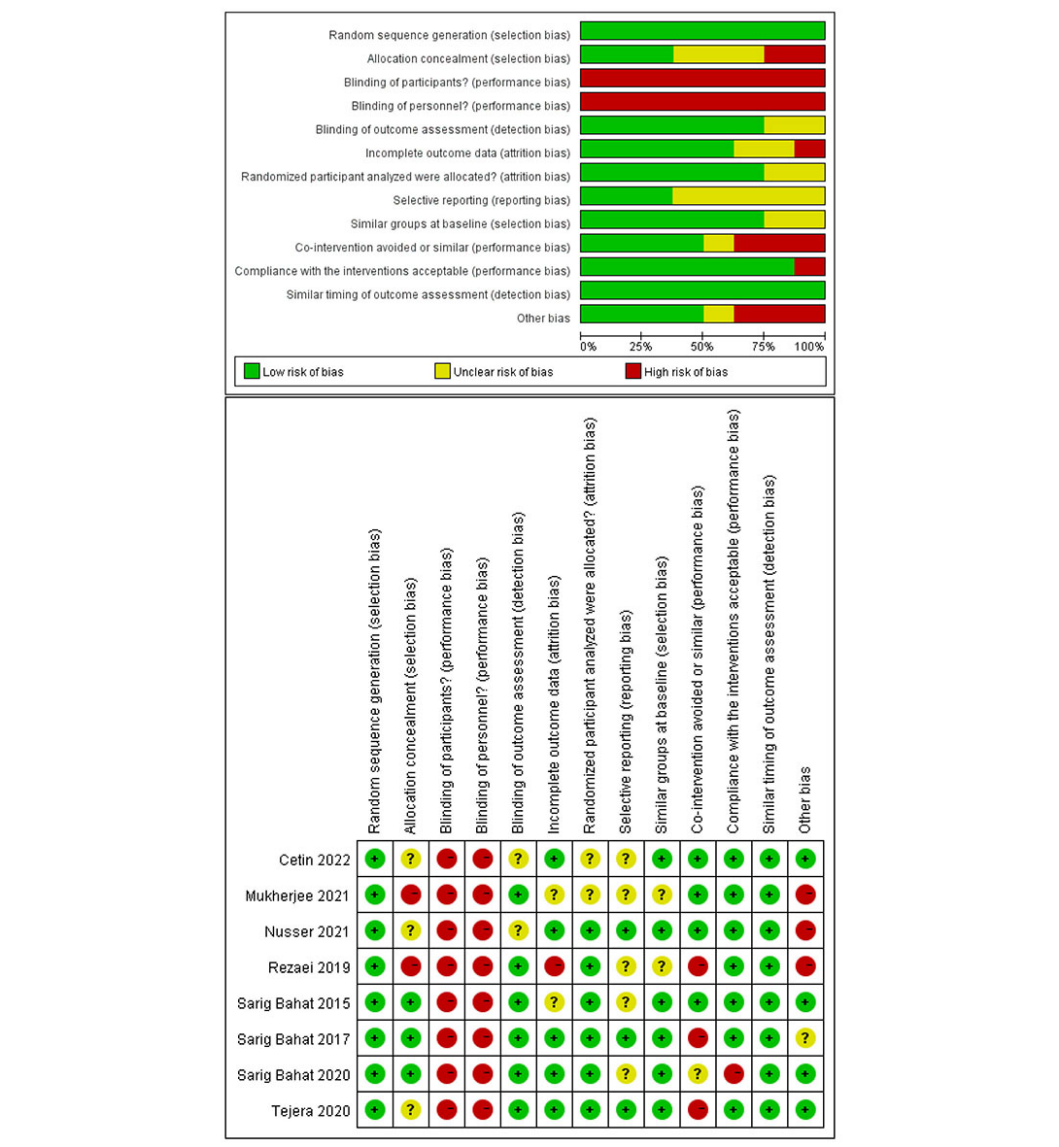
Summary of the quality evaluation and risk of bias in the included studies.

The overall risk of bias assessment results is shown in Figure 2. All (8/8, 100%) included studies did not obtain a high risk of bias in random sequence generation, blinding of outcome assessment, allocated analysis, selective reporting, baseline comparison, and measuring time point. In addition, 3 (38%) studies [38,42,43] reported allocation concealment, while 4 (50%) [39,40,42,43] addressed whether to collect follow-up data. A similar cointervention was satisfied in 4 (50%) studies [37,38,40,44], and 7 (88%) studies [37-42,44] revealed great compliance with the intervention. However, none of the included studies met the criteria of therapist or participant masking due to the nature of the VR intervention.

Based on the GRADE approach, we found moderate or high levels of evidence regarding pain intensity (overall, NRS, multimodal intervention, and chronic neck pain), disability, kinesiophobia, CROM, mean velocity, and peak velocity. These results suggested that the actual effect was likely close to the estimation. Moreover, the quality of evidence for the follow-up effect on pain intensity and disability was classified as very low, indicating that the actual effect may differ substantially from the estimates. The assessment details by the GRADE criteria are presented in Multimedia Appendix 2. Agreement between the authors was 100% at each stage.

### Effects of VR Therapy in Reducing Pain Intensity

**Figure 3:**
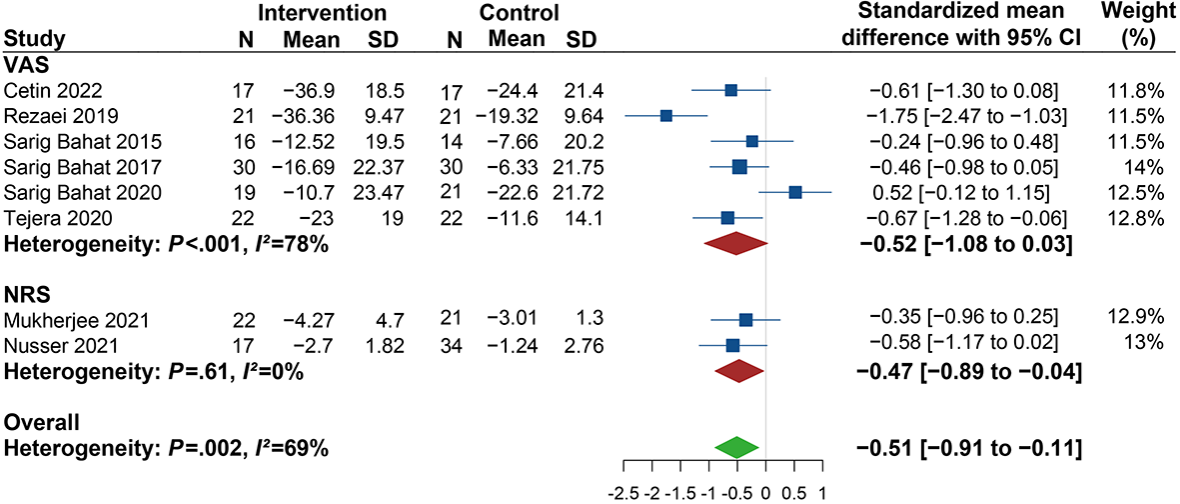
Forest plot of the effectiveness of virtual reality (VR) therapy in reducing pain intensity. NRS: numeric rating scale; VAS: visual analog scale.

The effect of VR therapy on reducing pain was evaluated by comparing the changes in pain intensity of the VR group and the control. As shown in Figure 3, the randomized effect model revealed a statistically significant decrease in pain intensity favored VR intervention compared to controls (SMD −0.51, 95% CI −0.91 to −0.11). Given the significant heterogeneity observed (*I^2^*=69%), we performed subgroup analyses to investigate the source of heterogeneity based on the different interventions, stages of neck pain, clinical operational model, and measuring tools.

**Figure 4:**
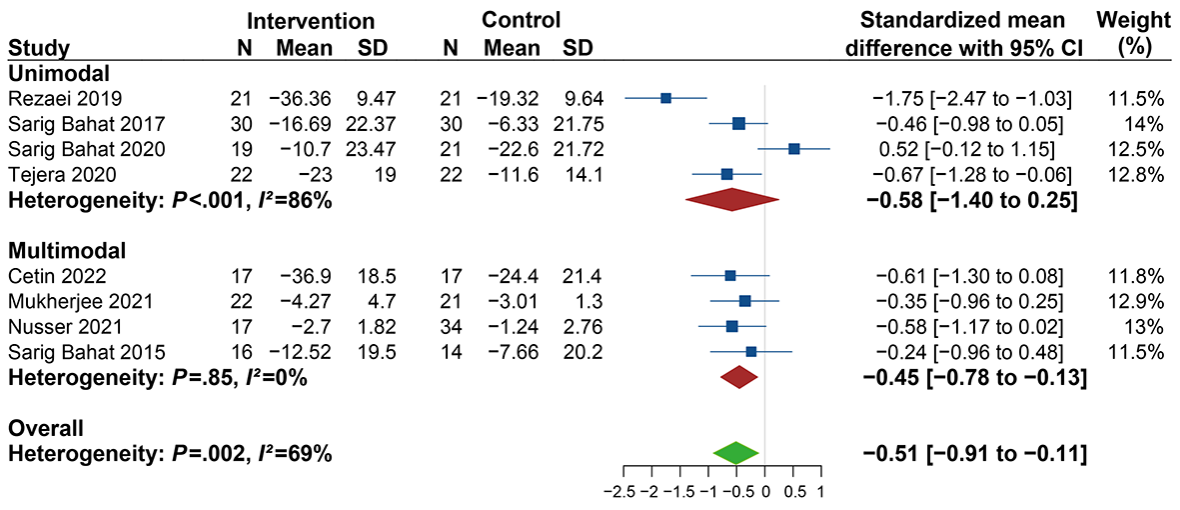
Forest plot of the effectiveness of different virtual reality (VR) interventions in reducing pain intensity.

In Figure 4, the results show significant differences (SMD −0.45, 95% CI −0.78 to −0.13) in pain intensity in favor of the multimodal intervention (VR therapy in combination with any other interventions) compared to other interventions, and no heterogeneity was found (*I*^2^=0%). When unimodal intervention (VR therapy alone) was compared with other therapies, the subgroup analysis showed no significant differences (SMD −0.58, 95% CI −1.40 to 0.25; *I*^2^=86%). These results show that the heterogeneity was mainly derived from the studies using unimodal intervention, indicating that multimodal intervention had a better impact on reducing pain intensity than unimodal intervention.

**Figure 5:**
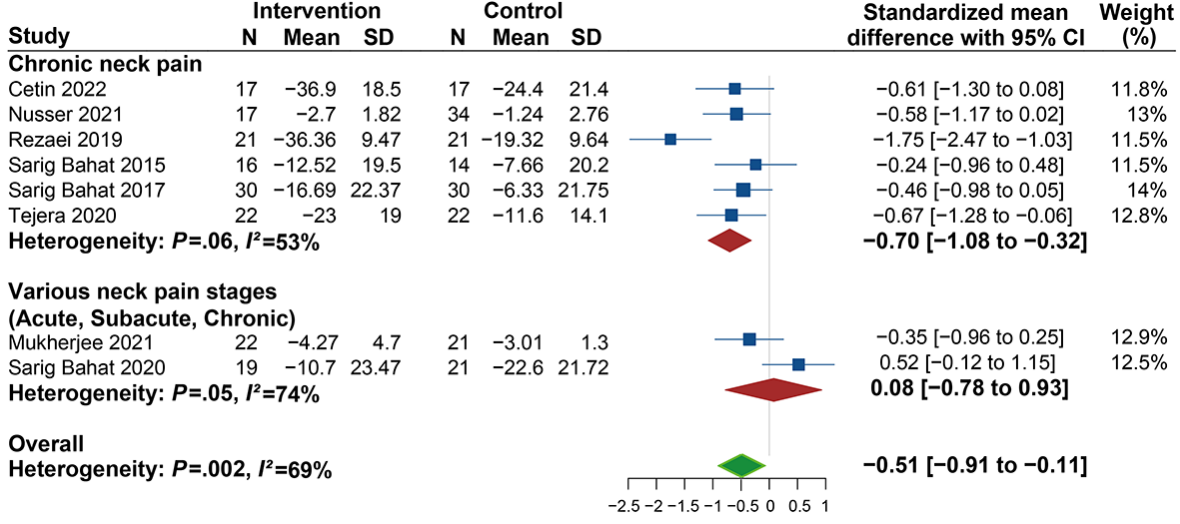
Forest plot of the effectiveness of virtual reality (VR) therapy for different stages of pain.

**Figure 6:**
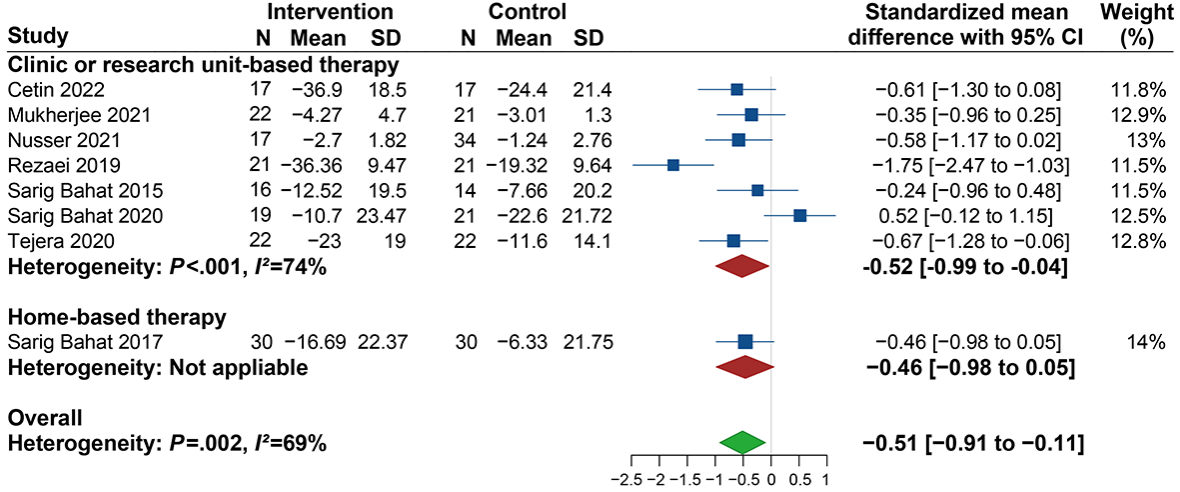
Forest plot of the effectiveness of virtual reality (VR) therapy in different clinical operational models.

In terms of the stages of neck pain, the significant decrease in pain intensity in the VR group was 0.70 lower than that in the control group (SMD −0.70, 95% CI −1.08 to −0.32; *I^2^*=53%) for patients with chronic neck pain. However, for patients with various stages of neck pain, no significant changes were found (SMD 0.08, 95% CI −0.78 to 0.93; *I*^2^=74%) (Figure 5). Regarding the measuring tools, the results show significant changes in pain intensity in studies investigating neck pain with an NRS (SMD −0.47, 95% CI −0.89 to −0.04) with no heterogeneity (*I^2^*=0%). However, no significant differences were observed in studies using VAS (SMD −0.52, 95% CI −1.08 to 0.03; *I^2^*=78%) (Figure 3). Meanwhile, significant improvements were revealed in the clinic or research unit–based therapy (SMD −0.52, 95% CI −0.99 to −0.04; *I^2^*=74%) but not found in the home-based therapy (SMD −0.46, 95% CI −0.98 to 0.05) subgroup (Figure 6).

**Figure 7:**
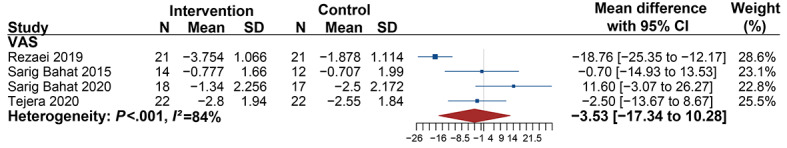
Forest plot of the follou-up effects of VR therapy in reducing pain intensity. VAS: visual analog scale.

In addition, we found no statistical difference in pain intensity at follow-up between the VR group and the control group (SMD −3.53, 95% CI −17.34 to 10.28; *I^2^*=84%). Due to the limitations of eligible literature, no subgroup analysis could be carried out (Figure 7).

### Effects of VR Therapy on Other Related Health Outcomes

As shown in Table 2, patients in the VR group had less disability (SMD −3.23, 95% CI −4.32 to −2.14; *I*^2^=46%)[38-42], lower kinesiophobia (SMD −0.30, 95% CI −0.59 to −0; *I^2^*=0%) [37-39,42], greater CROM (SMD 0.21, 95% CI 0.08-0.33; *I^2^*=35%) [37,38,40,42,44], greater cervical mean velocity (SMD 8.98, 95% CI 2.91-15.06; *I^2^*=46%) [38,42], and peak velocity (SMD 10.24, 95% CI 1.28-19.15; *I^2^*=39%) [38,42] compared to the control group. As for the follow-up effect on disability, we found no significant difference between the VR group and the control group (SMD −3.07, 95% CI −6.57 to 0.43; *I^2^*=67%) [38,39,41]. Considering the high heterogeneity across studies, no subgroup analysis was performed.

**Table 2:** Evaluation of virtual reality (VR) therapy for other health outcomes.

Variables	Studies, n	Patients, n	Effect size	95% CI	*P* value	*I* ^2^
NDI^a^	5	211	−3.23	−4.32 to −2.14	<.001	46%
NDI (follow-up)	3	112	−3.07	−6.57 to 0.43	.09	67%
TSK^b^	4	177	−0.30	−0.59 to −0.00	.05	0%
CROM^c^	5	1018	0.21	0.08 to 0.33	.05	35%
Mean velocity	2	352	8.98	2.91 to 15.06	.004	46%
Peak velocity	2	352	10.24	1.28 to 19.15	.03	39%

^a^NDI: Neck Disability Index.

^b^TSK: Tampa Scale of Kinesiophobia.

^c^CROM: cervical range of motion.

### Safety

Adverse events were reported in 2 (25%) studies [38,42]. In an RCT performed by Sarig Bahat et al [38], 10% (4/140) of participants experienced motion sickness during VR assessment, but no pain exacerbation was reported. In another study [42] conducted by the same authors, 6% (5/90) of the participants quit due to simulator sickness and headache during training, which may be attributed to the need for a high immersion level of VR devices. The remaining studies reported no evidence of adverse events.

## Discussion

### Overview

This systematic review investigated the effectiveness of VR therapy in treating patients with neck pain. The results, with a moderate level of evidence, indicated that VR was a beneficial nonpharmacological approach to pain management. The results of subgroup analyses showed that multimodal intervention had a better therapeutic effect on reducing pain intensity, whereas unimodal intervention did not have obvious effects. For different stages of pain, subgroup analyses indicated that VR could effectively relieve chronic neck pain. Based on the clinical operational model, patients treated in the clinic or research unit reported better outcomes than home-based rehabilitation. Regarding the other health indicators, VR therapy significantly alleviated disability, reduced kinesiophobia, and improved CROM and mean and peak velocity. Nevertheless, the follow-up effects of VR therapy on reducing pain intensity and disability were not found.

### Methodological Considerations

Among the risk factors of the CBN tool, various items of the selection, detection, and attrition bias in this systematic review were well controlled, as presented in Figure 2. The low level of bias in these 3 aspects indicates that the included RCTs may have effectively collected, evaluated, and analyzed the data, improving the generalizability and reliability of their findings.

However, a potential primary source of bias in the included studies was that none of the eligible studies achieved the blinding of participants or personnel due to the nature of the proposed VR therapy. The results might be inevitably influenced by these performance biases. There are currently no studies setting standard control groups to eliminate the placebo effect, which requires more attention from future researchers. Moreover, only 38% (3/8) of the studies reported outcomes in a prespecified manner. This unclear reporting bias may produce misleading results due to the selective outcomes reporting, so we recommend that readers treat our results more cautiously.

### Discussions of Results

In terms of pain management, studies using unimodal programs better represented the individual effects of VR therapy, whereas the multimodal approach may be consistent with the clinical scenario. The results of subgroup analyses revealed that the multimodal approach had a better analgesic effect than the control, while no significant difference was found in the unimodal intervention. Our conclusions are consistent with those of earlier studies [33,52]. These results might be explained by the effectiveness of VR in distracting attention and improving neuromuscular control [53,54]. However, the add-on effect of the multimodal intervention may exaggerate the actual therapeutic effect of VR therapy. Therefore, the individual analgesic effect of VR is still unclear.

Besides intervention, subgroup analysis indicated the beneficial effect of VR on patients with chronic neck pain, which was in line with the findings of previous studies [41,42]. Alterations of sensorimotor control were identified in many patients with chronic neck pain and were considered to play an important role in neck disability and limited motor function [55,56]. However, Rezaei et al [41] reported that VR effectively improved pain due to improved coordination between the deep and superficial cervical muscles. Enhanced coordination could better support the cervical segments and unload the stress on cervical structures to relax the neck and relieve pain, which was also confirmed by the therapeutic effects of VR therapy on cervical kinematic indicators (eg, CROM, mean and peak velocity) in this review. However, as a type of pain that lasts longer than 3 months, the lasting analgesic effect on chronic neck pain was a common problem in related research. In this review, no statistical difference at follow-up between the VR group and the control was found, limiting the subsequent clinical application of VR therapy.

As for the measuring tool, the results demonstrated that studies using an NRS showed a significant improvement in pain intensity, but no significant difference was observed in studies using a VAS. This conclusion is different from that of 2 earlier studies [33,57]. We speculated that this might be due to the different measuring sensitivities of the 2 scales on pain measurement. Regarding the clinical operational model, our findings supported the benefits of clinic or research unit–based therapy. As compared to home-based therapy, clinical or research unit–based therapy allowed for real-time supervision of patients by therapists to ensure the completeness and accuracy of the VR treatment, which probably contributed to a better recovery response. However, the actual therapeutic effect of VR therapy across different operational models remains elusive because of the relatively scarce literature and high heterogeneity across studies.

Apart from pain intensity, VR therapy was shown to be effective in improving other health-related indicators (disability, kinesiophobia, CROM, and mean and peak velocity). For the NDI score representing neck disability, our data suggested a beneficial effect of VR compared with other therapeutic methods, which is consistent with previous research [38,41]. We propose that this might be due to the pain relief, which allowed patients to perform more activities of daily living (ADL) involving cervical movement, resulting in an increase in CROM and velocity. Meanwhile, VR treatment significantly relieved kinesiophobia in patients due to the effectiveness of VR therapy in distracting attention, thus reducing disability and removing limitations on cervical movement.

However, the lasting therapeutic effects of VR on neck pain and disability were not found. This might be because the intervention period was short, and treatment effects could not be shown. Among the included studies, participants in 4 (50%) studies [37,38,40,43] received an overall VR intervention duration lower than 180 minutes, while 3 (38%) studies [39,42,44] allowed participants a total VR treatment time ranging between 180 minutes and 360 minutes. Such low treatment dosages might lead to unsustainable therapeutic effects of VR intervention. Therefore, more high-quality studies with long-term VR intervention on neck pain are required to investigate its sustained efficacy.

### Study Limitations and Implications

This study has a few limitations to consider. First, only 8 studies comprising a total of 382 patients were eligible for the meta-analysis. The heterogeneity between the included literature inevitably yields methodological errors and potential bias given the limited amount and different research designs of the included studies. Second, the conclusion of this systematic review should be treated with caution due to blinding limitations, although it was deemed very difficult to conduct blinding on therapists or participants due to the nature of the VR intervention. Third, psychological (eg, anxiety, depression, or stress) and health-related outcome (eg, ADL or quality of life) were lacking in this review. These outcomes were not found in most of the included studies but should be evaluated in future studies. (4) Only 4 (50%) studies [38,39,42,43] investigated the follow-up data over 3 months or more, indicating a lack of assessments on the lasting therapeutic effect of VR in patients with neck pain.

Currently, the COVID-19 pandemic can be an impetus for driving the adoption of VR in telemedicine to meet new requirements from public health measures to mitigate COVID-19 transmission [58,59]. However, due to the operational complexity and high expenditure of current VR therapy, the shuffling card or lighthouse model may be more appropriate for subsequent clinical applications [60,61]. Patients with neck pain can receive individual assessment and treatment in the community and provide real-time feedback to specialists for plan adjustment. These approaches help improve the efficiency of health care workers, reducing the risk of outbreaks, and enabling patients to access suitable therapies with efficacy monitoring, which are likely to be the future application scenario [62]. The findings of this study may provide some guidance and inspire researchers to perform more studies and clinical applications in this field.

Nonetheless, there is still insufficient evidence investigating the benefits of VR therapy on neck pain. The efficacy of VR intervention with different levels of immersion, desired intervention parameters, and common adverse events is uncertain. These issues have been illustrated in previous studies [15,63] and need to be refined in more well-designed trials, with a focus on larger sample sizes and longer interventions to facilitate the development of clinical guidelines. Further, researchers should classify the inclusion criterion for eligible trials and conduct a suitable experimental design for blinding therapists and participants to reduce bias. In addition, more comprehensive evaluation indicators (eg, psychological function or health-related quality of life) should be explored to reflect the effectiveness of VR therapy in all dimensions of health.

### Conclusion

There is moderate-quality evidence that VR therapy is a beneficial nonpharmacological approach to improve pain intensity in patients with neck pain, with advantages to multimodal intervention, patients with chronic neck pain, and clinic or research unit–based VR therapy. However, due to the high heterogeneity across the included RCTs, more robust future RCTs are required to yield firmer conclusions.

## Appendix

Multimedia Appendix 1 Search strategy.

Multimedia Appendix 2 Grading of Recommendations Assessment, Development, and Evaluation (GRADE) results.

## Abbreviations

ADL: activities of daily living
CBN: Cochrane Back and Neck
CROM: cervical range of motion
GRADE: Grading of Recommendations Assessment, Development, and Evaluation
MeSH: Medical Subject Headings
NDI: Neck Disability Index
NRS: numeric rating scale
PICO: Participants, Interventions, Control, and Outcomes
PRISMA: Preferred Reporting Items for Systematic Reviews and Meta-analyses
RCT: randomized controlled trial
SMD: standardized mean difference
VAS: visual analog scale
VR: virtual reality
