# Supplementary material for: Virtual Reality Intervention for Patients With Neck Pain: Systematic Review and Meta-analysis of Randomized Controlled Trials
Source: J Med Internet Res. 2023 Apr 3;25:e38256. doi: 10.2196/38256 (PMC10131665; doi:10.2196/38256)
Supplement: Multimedia Appendix 1 [file jmir_v25i1e38256_app1.docx]

Search Strategy.

1.Database: MEDLINE (via PubMed, to October 2022).

#1 neck pain [mh] or neck ache [tiab] or cervical spondylosis [tiab] (10288)

#2 virtual reality [mh] or Virtual Reality Exposure Therapy [mh] or VR [tiab] or virtual reality simulator [tiab] or virtual reality system [tiab] or telerehabilitation [mh] or remote rehabilitation [tiab] or virtual reality head-mounted display [tiab] (16273)

#3 #1 AND #2. (21)

2.Database: EMBASE (to October 2022).

#1 'neck pain'/exp OR 'cervical spondylosis'/exp OR 'neck ache':ab,ti (32419)

#2 'virtual reality exposure therapy'/exp OR 'virtual reality'/exp OR 'virtual reality simulator'/exp OR 'virtual reality system'/exp OR 'virtual reality head mounted display'/exp OR 'telerehabilitation'/exp OR 'remote rehabilitation':ab,ti OR 'vr':ab,ti (37351)

#3 #1 AND #2. (74)

3.Database: CENTRAL (to October 2022).

#1 MeSH descriptor: [Neck Pain] explode all trees (1591)

#2 TI ('neck pain' or 'neck ache' or 'cervical spondylosis') OR AB ('neck pain' or 'neck ache' or 'cervical spondylosis') (680)

#3 #1 OR #2 (2254)

#4 MeSH descriptor: [Virtual Reality Exposure Therapy] explode all trees (230)

#5 MeSH descriptor: [Virtual Reality] explode all trees (544)

#6 MeSH descriptor: [Telerehabilitation] explode all trees (182)

#7 TI ('virtual reality simulator' or 'virtual reality system' or 'virtual reality head-mounted display' or 'remote rehabilitation' or 'VR') OR AB ('virtual reality simulator' or 'virtual reality system' or 'virtual reality head-mounted display' or 'remote rehabilitation' or 'VR') (293)

#8 #4 OR #5 OR #6 OR #7 (1209)

#9 #3 AND #8 (30)

4.Database: Web of Science core collection (to October 2022).

#1 ((TS=('neck pain' OR 'neck ache' OR 'cervical spondylosis')) OR (TI=('neck pain' OR 'neck ache' OR 'cervical spondylosis')) OR (AB=('neck pain' OR 'neck ache' OR 'cervical spondylosis'))) (32905)

#2 ((TS=('virtual reality exposure therapy' OR 'virtual reality' OR 'virtual reality simulator' OR 'virtual reality system' OR 'virtual reality head-mounted display' OR 'remote rehabilitation' OR 'telerehabilitation' OR 'VR')) OR (TI=('virtual reality exposure therapy' OR 'virtual reality' OR 'virtual reality simulator' OR 'virtual reality system' OR 'virtual reality head-mounted display' OR 'remote rehabilitation' OR 'telerehabilitation' OR 'VR')) OR (AB=('virtual reality exposure therapy' OR 'virtual reality' OR 'virtual reality simulator' OR 'virtual reality system' OR 'virtual reality head-mounted display' OR 'remote rehabilitation' OR 'telerehabilitation' OR 'VR'))) （84871）

#3 #1 AND #2. (106)

5.Database: Physiotherapy Evidence Database (PEDro) (to October 2022).

1. virtual reality.

2. neck pain.

3. 1 AND 2. （12）

6.Database: SCOPUS (to October 2022).

#1 TITLE-ABS ( "neck pain" OR "neck ache" OR "cervical spondylosis" ) (15802)

#2 TITLE-ABS ( "virtual reality exposure therapy" OR "virtual reality" OR "virtual reality simulator" OR "virtual reality system" OR "virtual reality head-mounted display" OR "remote rehabilitation" OR "telerehabilitation" OR "VR" ) (85806)

#3 #1 AND #2. (55)

7.Database: ClinicalTrial (to October 2022).

#1 'neck pain' OR 'neck ache' OR 'cervical spondylosis' (1026)

#2 'virtual reality exposure therapy' OR 'virtual reality' OR 'virtual reality simulator' OR 'virtual reality system' OR 'virtual reality head-mounted display' OR 'remote rehabilitation' OR 'telerehabilitation' OR 'VR' (2278)

#3 #1 AND #2. (21)

8.Database: CNKI (to October 2022).

#1 （主题：颈痛）OR（主题：颈椎病）OR（主题：颈部疼痛） （47077）

#2 （主题：虚拟现实技术）OR（主题：虚拟现实）OR（主题：远程康复） (50869)

#3 #1 AND #2. (9)

9.Database: WANFANG (to October 2022).

#1 主题:(颈痛) or 主题:(颈椎病) or 主题:(颈部疼痛) （62979）

#2 主题:(虚拟现实) or 主题:(虚拟现实技术) or 主题:(远程康复) (60350)

#3 #1 AND #2. (6)
