# Supplementary material for: Virtual Reality Intervention for Patients With Neck Pain: Systematic Review and Meta-analysis of Randomized Controlled Trials
Source: J Med Internet Res. 2023 Apr 3;25:e38256. doi: 10.2196/38256 (PMC10131665; doi:10.2196/38256)
Supplement: Multimedia Appendix 2 [file jmir_v25i1e38256_app2.docx]

| **Certainty assessment** | | | | | | **Number of participants** | | **Effect** | **Certainty** |
| --- | --- | --- | --- | --- | --- | --- | --- | --- | --- |
| **№ of study** | **Risk of bias** | **Inconsistency** | **Indirectness** | **Imprecision** | **Other considerations** | **Exp.** | **Con.** | **Absolute (95% CI)** |  |
| **Pain intensity** | | | | | | | | | |
| 8 | not serious | serious ^a^ | not serious | not serious | none | 164 | 180 | SMD **0.51 SD lower** (0.91 lower to 0.11 lower) | ⨁⨁⨁◯ MODERATE |
| **Pain intensity -- NRS** | | | | | | | | | |
| 2 | not serious | not serious | not serious | serious ^b^ | none | 39 | 55 | SMD **0.47 SD lower** (0.89 lower to 0.04 lower) | ⨁⨁⨁◯ MODERATE |
| **Pain intensity -- Multimodal intervention** | | | | | | | | | |
| 4 | serious | not serious | not serious | not serious | none | 72 | 86 | SMD **0.43 SD lower** (0.81 lower to 0.05 lower) | ⨁⨁⨁◯ MODERATE |
| **Pain intensity -- Chronic neck pian** | | | | | | | | | |
| 6 | not serious | serious ^a^ | not serious | not serious | none | 123 | 128 | SMD **0.70 SD lower** (1.08 lower to 0.32 lower) | ⨁⨁⨁◯ MODERATE |
| **Pain intensity -- Clinic or research unit-based therapy** | | | | | | | | | |
| 7 | not serious | serious ^a^ | not serious | not serious | none | 134 | 150 | SMD **0.52 SD lower** (0.99 lower to 0.04 lower) | ⨁⨁⨁◯ MODERATE |
| **Pain intensity -- follow-up effect** | | | | | | | | | |
| 4 | serious | serious ^a^ | not serious | serious ^b^ | none | 75 | 72 | SMD **3.53 SD lower** (17.34 lower to 10.28 higher) | ⨁◯◯◯  VERY LOW |
| **Disability** | | | | | | | | | |
| 5 | serious | not serious | not serious | not serious | none | 106 | 105 | MD **3.23 SD lower** (4.32 lower to 2.14 lower) | ⨁⨁⨁◯ MODERATE |
| **Disability -- follow-up effect** | | | | | | | | | |
| 2 | serious | serious ^a^ | not serious | serious ^b^ | none | 57 | 55 | MD **3.07 SD lower** (6.57 lower to 0.43 higher) | ⨁◯◯◯  VERY LOW |
| **Kinesiophobia** | | | | | | | | | |
| 4 | not serious | not serious | not serious | not serious | none | 90 | 87 | SMD **0.30 SD lower** (0.59 lower to 0.00 higher) | ⨁⨁⨁⨁ HIGH |
| **Cervical range of motion** | | | | | | | | | |
| 5 | not serious | not serious | not serious | not serious | none | 486 | 532 | SMD **0.21 SD higher** (0.08 higher to 0.33 higher) | ⨁⨁⨁⨁ HIGH |
| **Cervical mean velocity** | | | | | | | | | |
| 2 | serious | not serious | not serious | not serious | none | 184 | 168 | SMD **8.98 SD higher** (2.91 lower to 15.06 higher) | ⨁⨁⨁◯ MODERATE |
| **Cervical peak velocity** | | | | | | | | | |
| 2 | serious | not serious | not serious | not serious | none | 184 | 168 | SMD **10.24 SD higher** (1.28 lower to 19.15 higher) | ⨁⨁⨁◯ MODERATE |

**CI:** Confidence interval; **MD:** Mean difference; SMD: Standardized mean difference

**Explanations**

a. Downgraded one level for serious inconsistency: the I^2^ value is greater than 50%

b. Downgraded one level for serious imprecision: fewer than 150 participants

**GRADE Working Group grades of evidence
High quality:** We are very confident that the true effect lies close to that of the estimate of the effect.
**Moderate quality:** We are moderately confident in the effect estimate: The true effect is likely to be close to the estimate of the effect, but there is a possibility that it is substantially different.
**Low quality:** Our confidence in the effect estimate is limited: The true effect may be substantially different from the estimate of the effect.

**Very low quality:** We have very little confidence in the effect estimate: The true effect is likely to be substantially different from the estimate of effect.
